# Genome Mining as an Alternative Way for Screening the Marine Organisms for Their Potential to Produce UV-Absorbing Mycosporine-like Amino Acid

**DOI:** 10.3390/md20080478

**Published:** 2022-07-26

**Authors:** Nedeljka Rosic

**Affiliations:** 1 Faculty of Health, Southern Cross University, Gold Coast, QLD 4225, Australia; nedeljka.rosic@scu.edu.au; 2 Marine Ecology Research Centre, Southern Cross University, Lismore, NSW 2480, Australia

**Keywords:** mycosporine-like amino acids, mycosporine-like amino acid biosynthesis, ultraviolet-absorbing compounds, sunscreen, omics, genome mining, bioactivity, bioproduct screening, biosynthetic gene clusters

## Abstract

Mycosporine-like amino acids (MAAs) are small molecules with robust ultraviolet (UV)-absorbing capacities and a huge potential to be used as an environmentally friendly natural sunscreen. MAAs, temperature, and light-stable compounds demonstrate powerful photoprotective capacities and the ability to capture light in the UV-A and UV-B ranges without the production of damaging free radicals. The biotechnological uses of these secondary metabolites have been often limited by the small quantities restored from natural resources, variation in MAA expression profiles, and limited success in heterologous expression systems. Overcoming these obstacles requires a better understanding of MAA biosynthesis and its regulatory processes. MAAs are produced to a certain extent via a four-enzyme pathway, including genes encoding enzymes *dehydroquinate synthase*, *enzyme O-methyltransferase*, *adenosine triphosphate grasp*, and *a nonribosomal peptide synthetase*. However, there are substantial genetic discrepancies in the MAA genetic pathway in different species, suggesting further complexity of this pathway that is yet to be fully explored. In recent years, the application of genome-mining approaches allowed the identification of biosynthetic gene clusters (BGCs) that resulted in the discovery of many new compounds from unconventional sources. This review explores the use of novel genomics tools for linking BGCs and secondary metabolites based on the available omics data, including MAAs, and evaluates the potential of using novel genome-mining tools to reveal a cryptic potential for new bioproduct screening approaches and unrevealing new MAA producers.

## 1. Introduction

Mycosporine-like amino acids (MAAs) are natural sunscreens and the most common group of secondary metabolites among aquatic organisms protecting them from harmful ultraviolet radiation (UVR) [1]. MAAs are discovered in various marine and freshwater species, including micro and macroalgae, cyanobacteria, and aquatic animals ranging from invertebrates to vertebrates [2,3,4,5,6,7,8,9]. These small hydrophilic compounds (<400 Da) are characterised by a core molecule made of cyclohexenone or cyclohexenimine rings accompanied by additional radical groups [10,11,12]. The combinations of different radical groups with the core molecule resulted in the identification of more than 30 different MAAs in nature [9,12,13,14]. MAAs are characterised by different UV properties and additional bioactivities (see Table 1) [2,11,15,16].

**Figure 1:**
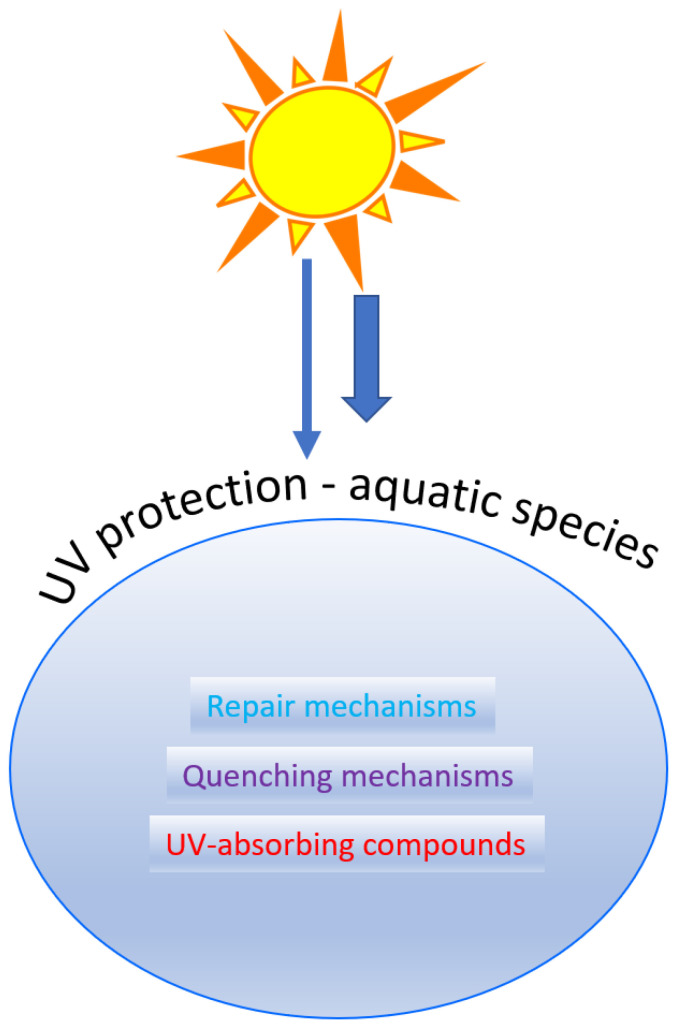
UV protection in aquatic species occurs at multiple levels, including UV-absorbing compounds (i.e., MAAs and other pigments) plus quenching mechanisms (i.e., enzymatic and non-enzymatic) and repair mechanisms (e.g., DNA repair).

In marine and freshwater organisms, UV protection occurs at multiple levels (Figure 1). Beyond UV-absorbing compounds conducting the UV screening, there are also additional mechanisms employed against damaging UVR, including quenching (i.e., enzymatic and non-enzymatic) and repair mechanisms (e.g., DNA repair) [30]. The synthesis of different UV-absorbing MAAs provides the main mechanism for efficient UV photoprotection (280–400 nm) [11]. MAAs also demonstrate additional pharmacologically relevant properties such as antioxidative, anti-inflammatory, and antiaging capacities [31]. In addition, MAAs were found to play a role in osmotic regulation and in cellular protection during drought periods [11,14,32,33,34]. MAAs can absorb light in the range of UV-A (315–400 nm), which makes ~95% of UV energy that penetrates the atmosphere and UV-B (280–315 nm) [12,35,36]. Prolonged sun exposure, specifically UVR, results in the production of free radicals and oxidative stress leading to potential damage to cellular structures and DNA [37]. MAA secondary metabolites reduce oxidative cellular damage due to their antioxidative properties and ability to scavenge free radicals [31,38]. The MAA UV-protective plasticity occurs due to the ability to produce various MAAs and to increase their quantities during the summer season under higher levels of UVR [31,36].

Antioxidants, isolated from natural resources or synthetically produced, are commonly used as bioactive compounds in modern medicine due to their ability to decrease the number of free radicals in cells and tissues [39]. MAA antioxidative properties are demonstrated via their ability to scavenge reactive oxygen species (ROS) and suppress singlet oxygen-induced DNA damage [21,31]. MAAs, such as porphyra-334 and shinorine, were found to increase the expression of NrF2 genes from the cytoprotective KeaP1/NrF2 pathway [21]. This pathway is important for synthesising proteins involved in maintaining cellular homeostasis and protection from oxidative stress [40]. In addition, MAAs accumulated in the cytoplasm of cyanobacteria play a role in osmotic regulation and drought stress protection [33]. MAAs also provide cellular protection from salt stress [41], while at the same time, they are used as a source of nitrogen, which is important for daily photosynthetic processes [42].

Beyond their UV-protective and antioxidative properties, MAAs demonstrate additional biotechnological potentials, including anti-inflammatory, anti-proliferative, and antiaging properties (Table 1) [14], that could be further explored for more generation of environmentally friendly sunscreen with advanced capacity for skin cancer prevention [10,43]. However, MAAs have been used in very limited ways in several cosmetic applications as natural agents for sun protection [44]. For example, porphyra-334 and shinorine isolated from red alga *Porphyra umbilicalis* have been used in the commercial product Helioguard^®^ 365, which was promoted for containing natural UV-screening compounds [45]. Helionori^®^ is another commercial product that protects from UVR and contains MAAs palythine, porphyra-334, and shinorine [44].

MAA synthesis occurring via the shikimate pathway [12] and/or the pentose phosphate pathway [46] was also analysed using genome-mining approaches [47,48]. The genes from the MAA pathway (called the *mys* cluster) were first isolated from the cyanobacterium *Anabaena variabilis* and contained four genes: *dehydroquinate synthase* (DHQS), *O-methyltransferase* (O-MT), *adenosine triphosphate* (ATP) *grasp,* and *nonribosomalpeptide synthetase* (NRPS) [46]. The variability in genes encoding enzymes important for the MAA biosynthesis pathways was confirmed using genomics data encompassing over 300 cyanobacteria [48] and microalgae *Symbiodiniaceae* [49]. Consequently, there are still substantial knowledge gaps in understanding MAA biosynthesis, which needs to be resolved and is important for enabling the use of MAAs in biotechnology, especially when applying heterologous expression systems [50]. Therefore, further genomic analyses are needed to fully elucidate differences in MAA biosynthetic gene clusters (BGCs) in various MAA-synthesis-capable species. This review discusses ways to improve MAA biosynthetic knowledge, including the application of genome-mining screening approaches in MAA discoveries.

## 2. Traditional vs. New Ways of Natural Products Discovery

**Figure 2:**
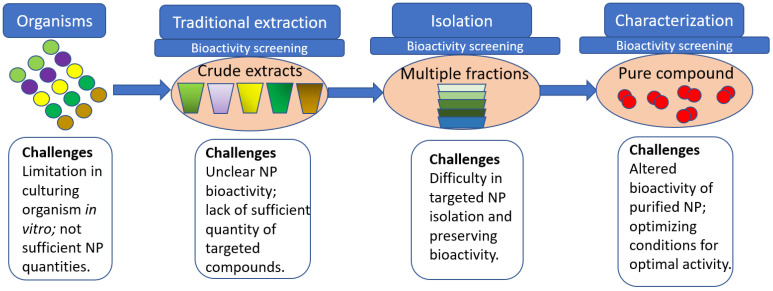
Traditional pathway in discovering natural products (NP) with bioactive properties, including challenges.

The emerging treatments of currently uncured diseases have mainly focused on exploring and utilising novel natural products (NPs) [51]. Therefore, the discovery of new NPs has been driven by the constant need for new compounds with supreme and improved bioactive properties, sustainable in vitro production, and a high yield of bioactive compounds [52,53,54]. These sustainability aspects of targeted NPs have been critical when assessing the biotechnological capacity of the bioactive compounds for potential pharmaceutical and industry applications [40,55]. Traditionally, the discovery pathways to the novel NPs were driven by the bioactivity-guided pathway that begins with the extraction step, followed by the isolation and purification of the molecule of interest (Figure 2). During these isolation and purification steps, the assessment of biological activities is happening in parallel, as the presence or absence of other molecules or external conditions may impact the activity of the targeted NP. These bioactivity screening options, which are often performed using different phenotypic assays, are implemented to confirm the presence of bioactivity in newly discovered NP [56,57,58]. However, the molecular mechanism of action for these NPs is often unclear; therefore, the application of specific phenotypic assays for revealing NPs actions (i.e., proteome profiling and other assays) is useful at this discovery stage [40,59]. Another obstacle in the traditional NP discovery pathway includes the purification efficiency for a targeted molecule and the availability of appropriate tools for chemical characterisation [40]. Furthermore, after successful NP isolation, characterisation, and confirmation of the beneficial bioactivity, additional limitations preventing its use in biotechnological applications may include the limited availability of natural resources [60] or culturing or mass production capacities [61,62]. Sustainable use of natural resources is currently on the list of priorities for policymakers worldwide to preserve ecosystems and protect biodiversity [63]. Anthropogenic pollution from chemicals such as agricultural pesticides, industrial chemicals, and air and water pollutants has been recognised as a global problem and an increased risk to humanity and environmental health [64,65]. Current issues important for environmental protection and preserving natural biodiversity could be partially resolved by the use of environmentally friendly, natural compounds in biotechnological applications [38,66].

During the last decade, there has been a shift towards new genomics-based NP discoveries that include applying next-generation sequencing data and new bioinformatics tools [55,67]. With a significant increase in the number of sequenced organisms provided within publicly available databases, genome-mining approaches can be used for unravelling the cryptic biosynthetic potential in species previously unidentified for the synthesis of targeted molecules and, consequently, for novel drug discoveries [68].

Recently, applying the traditional isolation and screening approach to red algae resulted in the identification of 23 potential MAAs in 40 different seaweed species from Brittany [69]. However, for an efficient application of MAAs in biotechnology, the key obstacles to be overcome include: (i) limited availability of MAAs from natural resources; (ii) the lack of clear understanding of the MAA biosynthesis; and (iii) inadequate success in MAA production in heterologous expression systems [43,44]. The widespread industry use of MAA was in some ways prevented due to limited success in heterologous MAA expression and also the low extraction yields from natural sources. The potential solution was investigated by the application of chemical synthesis, which resulted in the generation of synthetic MAA analogues [70]. However, these synthetic compounds only provide a very narrow range of pharmacological and UV-absorbing properties compared to the properties found in MAAs isolated from nature. Consequently, the existing limitations prompted new research directions via the application of big data and using novel omics approaches.

## 3. From Genes to Biotechnological Solutions

**Figure 3:**
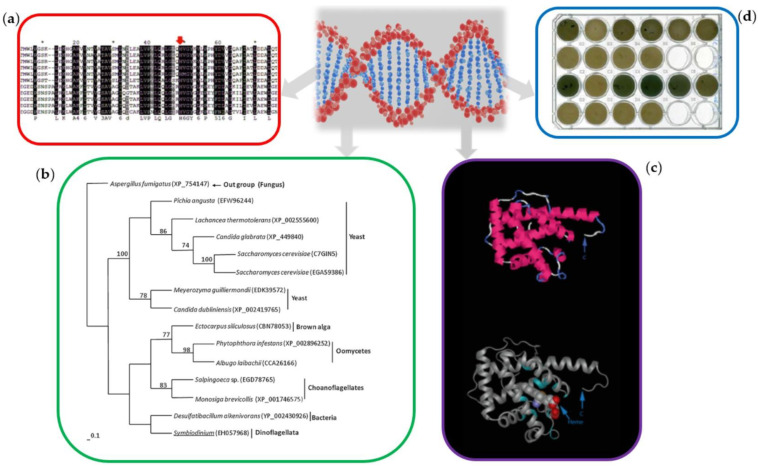
Genome mining and applications of omics data: (**a**) sequence analysis of haemoglobin-like genes obtained from microalgal transcriptomics data [73]; (**b**) phylogenetic analysis of MAA biosynthesis genes from the omics data [49]; (**c**) protein modelling of targeted haemoglobin protein from the omics data [73]; (**d**) functional analyses after gene sequence manipulation via molecular evolutionary in vitro and the creation of new cytochrome P450 enzymes [58,78,79].

By applying genomic and phylogenetics methodologies, the variability in BGCs for the synthesis of various secondary metabolites has been revealed in different species [71]. Novel genes such as Cytochrome P450 and haemogobin were unexpectedly recovered in transcriptomics data [72,73], with heat shock proteins demonstrating differences in genetic polymorphism and differential gene expression profiles (Figure 3) [74,75]. Structural analyses and protein modelling from discovered BGC allowed better anticipation of protein function and gene origin, like in the case of MAA biosynthetic clusters [49]. The MAA BGC were identified in the cyanobacterium, including MAA gene analogous to some other analysed species in their omics data [46]. Similarly, the genes from the *mys* cluster encoding enzymes for the MAA biosynthetic pathway [46] were identified in the transcriptomics data of symbiotic dinoflagellates, the *Symbiodiniaceae,* including genes and their homologs of *dehydroquinate synthase*, adenosine triphosphate (ATP) grasp, O-methyltransferase; *nonribosomal peptide synthetase* (NRPS)-like genes [49]. The microbial origin of these *mys* genes in symbiotic dinoflagellates was confirmed via phylogenetic analyses (Figure 3) [49]. These types of analyses applied the forward genetic approach, starting from the phenotype (i.e., established presence of targeted NPs) towards sequence analyses. In contrast, the novel reverse genetic approaches go the opposite way by analysing sequence data and predicting the synthesis capacity [51]. These new approaches for assessing genomics sequence data allowed further discoveries [76] and improved further by applying different algorithms and machine learning tools for BCG discovery [77].

One of the suggested ways to overcome the issue of limited bioavailability of natural resources includes culturing of certain organisms such as microalgae [53]. These photosynthetic microorganisms are easy to be cultivated in vitro, and using cyanobacterial farming can be applied for massive and sustainable production of desired compounds and energy while decreasing CO_2_ emission [80]. Biotechnological processes using in vitro cultures of *Escherichia coli* are still applied as heterologous expression systems for the manufacturing of numerous biopharmaceuticals [81] and recombinant proteins [82]. The application of heterologous expression systems in recombinant biotechnology is performed via the introduction of foreign biosynthetic gene clusters into the host genome of *E. coli*, yeast, or other suitable organisms [82,83]. Recombinant proteins are synthesised in different organisms, including bacteria, yeast, and other heterologous expression systems [50,82,83,84]. In *E. coli*, human genes were successfully expressed and even manipulated via DNA family shuffling methodology (i.e., molecular evolution in vitro) and analysed for the presence of improved catalytic properties in new mutant genes created [58,78,79], and evaluated in the screening process (Figure 3d).

The biosynthetic gene cluster encoding the synthesis of UV-absorbing MAAs was also successfully isolated from the cyanobacterium *Anabaena variabilis* ATCC 29413 and expressed in bacteria [46]. Specifically, in vitro, the synthesis of primary MAA, shinorine, was successfully implemented in *E. coli* after introducing the cyanobacterial 4-gene *mys* cluster [46]. MAAs were also heterologously expressed in *E. coli* using a 5-gene cluster (*mylA–E*) isolated from cyanobacterium *Cylindrospermum stagnale* and resulted in the synthesis of mycosporine-lysine and the new MAA mycosporine-ornithine [50]. An MAA gene cluster isolated from *Nostoc linckia* resulted in the production of MAA precursor 4-deoxygadusol, then four MAAs (i.e., mycosporine-glycine, porphyra-334, shinorine, mycosporine-glycine-alanine, and palythine) [84].

MAAs molecules as secondary metabolites that are well recognised for their role in molecular interactions, ecological function, and other protective roles are characterised by the low yield in natural resources [14]. The application of omics technologies opens the window for the simultaneous discovery of multiple compounds by unrevealing the genetic signature and hidden biosynthetic potential of certain organisms [10]. Application of various approaches, including metabolomic, transcriptomic, proteomic, even metatranscriptomics, metagenomics, and other multi-omics, speed up the NP discovery rate [55,67,85]. Metagenomics or meta-transcriptomics data, containing multiple species sequences, often host and related-symbionts, such as reef-building corals and their microbial symbionts, provide insight into the symbiosis functionality and response to changing environmental conditions [86,87,88,89].

Various omics approaches were recently reviewed for MAAs and other NPs regarding the use of next-generation of sequencing (NGS) data, assessment of biosynthetic pathways, phylogenetic analyses, and liquid chromatography-tandem mass spectrometry data [10,76,90]. Therefore, heterologous expression systems were employed in an attempt to produce certain types of MAAs [46]. Although the expression of specific MAAs was successful in *E. coli* and other systems, there was still limited success as only certain types of MAAs were heterologous expressions produced in vitro [46,47]. Cyanobacterial MAAs were heterologously expressed as a result of five gene cluster (*mylA* to *mylE)* in *E. coli,* resulting in the production of mycosporine-lysine and mycosporine-ornithine [50]. Using a bioinformatic approach, the MAA BCG was identified in *Nostoc* and expressed in *E. coli*, producing direct MAA precursor 4-deoxygadusol and five MAAs (mycosporine-glycine, porphyra-334, shinorine, mycosporine-glycine-alanine and palythine [84]). The MAAs BGC from two Actinomycetales species were found to be homologous to cyanobacterial BGC and, when heterologously expressed in the host *Streptomyces, new* mycosporine-glycine-alanine was produced [47]. Interestingly, when *Actinosynnema mirum* was maintained in culture, lack of MAA production indicated the cryptic state of BGC, which sometimes may prevent detection of MAAs. The cryptic BGCs present the clear challenge to discover the full potential of analysed organisms as activation clues may be missing, where the advantage of genome mining may solve and provide insights for new secondary metabolite discoveries [76].

## 4. Genome-Mining Tools

Analyses of genomic sequences provided a hidden glance into organisms’ potential for producing bioactive NPs. Modern strategies in genome mining include in silico methods for BGC identification to facilitate the discovery of novel NPs [91]. For genome mining, different types of sequence data (e.g., genomics, transcriptomics, metabolomics, proteomics, epigenomics, and multi-omics) [10,40,54] and numerous bioinformatics tools are applied [67,92]. During this process, the starting point is the accessibility of omics data from publicly available databases such as GenBank at the National Center for Biotechnology Information (NCBI) [93] and other integrated databases resources such as the International Nucleotide Sequence Database Collaboration (INSDC; http://www.insdc.org/, accessed on 12 June 2022) [94]. The majority of genome-mining strategies targeted the specific BGC encoding enzyme important for NP synthesis [76] using various computational tools. The overview of commonly used bioinformatics tools, especially applicable for microbial BGCs, is provided in Table 2.

The original targeted-based genome-mining approaches applied reference alignment using a basic local alignment search tool known as BLAST [116]. Later, rule-based algorithms with the comprehensive pipeline improved the identification of BGCs for secondary metabolites from bacterial and fungal sequence data using AntiSMASH (http://antismash.secondarymetabolites.org; accessed on 12 June 2022) [95] with additional updated versions for improved detection of related BGCs [96,97,98,99,100]. Another useful computational tool is PRISM, which uses microbial genomic data and identifies BGCs for nonribosomal peptides and some polyketides [105,106,107]. This genome-guided prediction tool includes the complete chemical structure for all currently used bacteria-driven antibiotics and also forecasts possible NPs based on cryptic BGCs [107]. Both AntiSMAH and PRISM apply multiple sequence alignment-based profile Hidden Markov Model (HMM) [77]. ClusterFinder uses the machine learning approach and HMM for the systematic identification of BGCs [117]. However, this computational tool has limited detection of the higher-order properties of BGCs (e.g., position dependency effects) [77,118]. The RiPPER genome-mining tool is used for the discovery of BGCs encoding specialised microbial metabolites, ribosomally synthesised, and post-translationally modified peptides (RiPPs) [110]. Due to a lack of shared pattern, in silico prediction of RiPP BGCs was performed by identification of the co-existence of a specific precursor peptide and RiPP tailoring enzymes, but this is possible only for the identification of already discovered RiPP families.

Further development of genome mining applied the use of a deep-learning approach, including the use of recurrent neural networks (RNNs) and the protein family database (http://pfam.xfam.org; accessed on 12 June 2022)) [119]. Another deep-learning strategy (DeepBGC) applied the BGC prediction algorithm to further improve the identification of de novo bacterial gene clusters (https://github.com/Merck/deepbgc; accessed on 12 June 2022) [77]. Open sources for the prediction of BGC, such as Prodigal (http://compbio.ornl.gov/prodigal/; accessed on 12 June 2022) and automated annotation, helped in reducing false-positive BGCs identification [120]. Genome mining search for novel antibiotics, Antibiotic-Resistant Target Seeker (ARTS), is available at https://arts.ziemertlab.com (accessed on 12 June 2022) [121]. Recently, a new machine learning approach, GECCO (GEne Cluster prediction with COnditional random fields; https://gecco.embl.de; accessed on 12 June 2022), allowed much higher identification of de novo BGCs from metagenomics data, confirming the important link for prediction between protein domain and secondary metabolites [91]. An overview of different bioinformatics tools applied for genome mining and useful in the discovery of BCGs and related secondary metabolites are presented in Table 2. All these different genome-mining approaches allowed the unexpected discovery of important genes or gene clusters within unforeseen organisms.

The discovery of MAA biosynthetic capacities has been traditionally guided by phenotype or conformation of MAA profiles after chemistry-guided extraction (Figure 2). Follow-up genomic tools have been used via the application of traditional BLAST genome-mining approaches [48,49] for a better understanding of MAA biosynthetic pathways [46,47]. However, genotype-driven MAA discoveries via the application of diverse genomics tools such as the one presented in Table 2 are yet to be efficiently utilised. Using various genome-mining tools incorporating machine learning [117] and a deep-learning approach [119] will allow genotype-driven MAA discoveries or the discovery of a cryptic potential for MAA biosynthesis from currently unidentified resources. Therefore, applying advanced genome-mining tools could help overcome current biotechnological obstacles limiting cost-effective MAA industry applications by extension of available natural resources and a better understanding of regulatory mechanisms involved in MAA synthesis.

## 5. Conclusions

MAAs are secondary metabolites with promising potential to be used as marine-derived sunscreens due to their supreme UV-absorbing capacity and additional pharmacologically beneficial properties. The application of modern bioinformatics tools in genome mining provides exciting opportunities for the characterisation of the MAA biosynthetic pathways, its expression patterns and regulatory mechanisms, which could overcome current existing obstacles in scaling up MAA production in vivo and in vitro. Furthermore, new/old MAA compounds can be rediscovered in completely new resources, providing ecologically friendly alternatives for preserving natural biodiversity. Applying genome mining will provide an alternative way of screening marine and other organisms for their potential to produce MAA. Consequently, this will unlock biotechnological potential for the use of MAAs in cosmetic, pharmaceutical, and other industries, including producing environmentally friendly organic sunscreens with therapeutic properties.

## Figures and Tables

**Table 1:** MAA precursor and primary MAAs, including their chemical structure, bioactivities, and UV-absorbing maximum.

MAA Name (Molecular Formula; Mw)	Chemical Structure	Key Features (ʎmax)/Bioactive Properties [Reference]
4-deoxygadusol (C_8_H_12_O_5_; 188 g/mol)	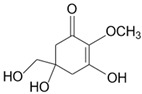	Direct MAA precursor UV-absorbing property (268 nm)
Mycosporine-glycine (C_10_H_15_NO_6_; 245 g/mol)	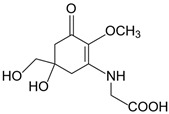	UV-absorbing property (310 nm) Antioxidative [17,18,19,20] Anti-inflammatory [18] Antiaging [18]
Shinorine (C_13_H_20_N_2_O_8_; 332 g/mol)	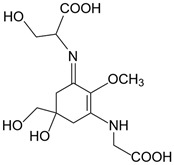	UV-absorbing property (333 nm) Antioxidative [17,19,21] Anti-inflammatory [18,22] Antiaging [18] Anti-adipogenic [23]
Porphyra-334 (C_14_H_22_N_2_O_8_; 346 g/mol)	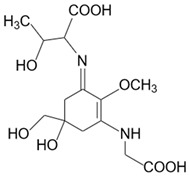	UV-absorbing property (334 nm) Antioxidative [17,19,21] Anti-inflammatory [22] Antiaging [24] Anti-adipogenic [23]
Mycosporine-2-glycine (C_12_H_18_N_2_O_7_; 302 g/mol)	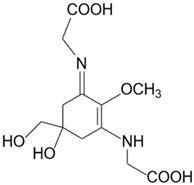	UV-absorbing property (332 nm) Antioxidative [19,25,26] Anti-inflammatory [26] Antiaging [26]
Palythine (C_13_H_20_N_2_O_5_; 284 g/mol)	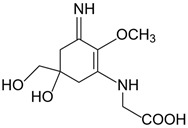	UV-absorbing property (320 nm) Antioxidative [25,27] Anti-proliferative [28] Antiaging [29]

**Table 2:** Genome-mining tools for identification and analyses of biosynthetic gene clusters (BGCs) relevant to the synthesis of secondary metabolites and other natural products (NPs).

Software Name (Key Features)	Website Availability	Application and Improvements	Reference
AntiSMASH (ANTIbiotics & Secondary Metabolite Analysis Shell; BCGs discovery in bacteria and fungi genome sequences)	Bacteria: antiSMASH bacterial version (secondarymetabolites.org/, accessed on 12 June 2022) Fungi: antiSMASH fungal version (secondarymetabolites.org, accessed on 12 June 2022)	Release of software	[95]
		Improved versions (2–5)	[96,97,98,99]
		The latest version (6) with improved BCGs detection	[100]
BAGEL (Automated identification of genes encoding ribosomally synthesised and post-translationally modified peptides -RiPPs)	Bacteria: http://bagel.molgenrug.nl http://bagel4.molgenrug.nl/, accessed on 12 June 2022	Release of software	[101]
		BAGEL2	[102]
		BAGEL3	[103]
		BAGEL4	[104]
PRISM (PRediction Informatics for Secondary Metabolomes; prediction of chemical structures of NPs)	Microbe genomes: http://magarveylab.ca/prism/, accessed on 12 June 2022	Release of software	[105]
		PRISM 3	[106]
		PRISM 4	[107]
CLUSEAN (CLUster SEquence Analyzer; bacterial secondary metabolite BCGs, automated analyses; Bioperl-based annotation pipeline)	Bacteria: https://bitbucket.org/tilmweber/clusean, accessed on 12 June 2022	Release of software	[108]
RiPPMiner (Automated Prediction of BGCs and Crosslinked Chemical Structures of -RiPPs)	http://www.nii.ac.in/rippminer.html, accessed on 12 June 2022	Release of software	[109]
RiPPER (for detection of BGCs of– RiPPs)	Actinobacteria: streptomyces/ripdock—Docker Image|Docker Hub	Release of software (Specific application for thioamidated ribosomal peptides) Updated version	[110] [111]
RODEO (Rapid ORF Description and Evaluation Online; for detection of BGCs for RiPPs)	http://www.ripp.rodeo/, accessed on 12 June 2022	Release of tool (AntiSMASH combined with Pfam * domain prediction)	[112]
BiG-SCAPE (The Biosynthetic Gene Similarity Clustering and Prospecting Engine; build sequence similarity report for new BGCs; using metabolomic data)	Multigenomes: BiG-SCAPE CORASON|July 2018 (secondarymetabolites.org, accessed on 12 June 2022)	Release of software	[113]
plantiSMASH (antiSMASH for plant genomes)	Plant genomes: http://plantismash.secondarymetabolites.org, accessed on 12 June 2022	Release of tool	[114]
PhytoClust (discovery of Metabolic Gene Clusters (MGCs) in plant genomes)	http://phytoclust.weizmann.ac.il/, accessed on 12 June 2022	Release of tool	[115]

* Pfam: Home page (xfam.org, accessed on 12 June 2022).
